# Supplementary material for: Association of lipoprotein (a) with coronary artery disease in a South Asian population: A case-control study
Source: PLoS One. 2022 May 3;17(5):e0267807. doi: 10.1371/journal.pone.0267807 (PMC9064091; doi:10.1371/journal.pone.0267807)
Supplement: Attachment — Submitted filename: To.docx [file pone.0267807.s002.docx]

To,

The Editor

PLOS ONE

Dear Prof. Katriina Aalto-Setala,

We have re-written the manuscript titled “Lipoprotein(a) as a risk factor for cardiovascular disease in a South Indian population: A case control study” (PONE-D-21-06053).

We would like to re-submit the revised version for consideration of publication.

We have addressed the comments of the reviewers given below.

Going in accordance with the reviewer’s comments:

Reviewer 1. : **In this study, the authors have analysed the largest sample in India and tested the association of Lp(a) levels between CAD cases and controls. The authors recommend that Lp(a) should be measured in a routine lab report and show how Lp(a) is significantly higher in cases <50yrs. Lipoprotein(a) has had tremendous coverage in recent ATVB (Jan 2021, Vol 41, Issue 1) and is very topical.**

**My main concern with this is the lack of comparison with LDL-C. If the authors can convincingly show that the LDL-C is not significantly associated with CAD between cases & controls (overall and <50yrs). Furthermore, show similar comparison plot of LDL-C with age and disease severity, then perhaps there will be greater enthusiasm and strength in this report.**

**Another point the authors themselves make is the presence of different methods to measure Lp(a) and that can cause some concern to labs that are not fully equipped to report "accurate standardized" Lp(a) measurement.**

**Minor:**

**Figure 1 (correlation): The plot is showing comparison and not really correlation!**

**I would have preferred to do the statistical tests in matched age/sex cases and control samples but I understand it is not always possible and hope the statistical model accounts for this mismatch.**

**I found the reasoning for performing the different statistical tests a bit thin and needs to be clarified.**

**Response:** As per the reviewer’s comments we have analysed LDL-Cholesterol values and compared the same between diseased and non-diseased for all age-groups and also for age< 50 years.

With regards to the uniformity of tests, the Lp(a) was assessed using the immune-turbidometric method and the same test kit (Randox) in both hospitals on the same model of automated analyser, Hitachi 907.

“Correlation” has been changed to “comparing”

The statistical analysis and results section has been re-worked on and explained in more detail.

**Reviewer 2. This a potentially valuable paper on higher Lp(a) as a risk factor for CAD in Indian patients.**

**The clinical outcome data are consistent with expectations. The angiographic data do not support the overall hypothesis.**

**The Introduction should be no more than 3 paragraphs. 2.5 pages is over-excessive with most of text redundant.**

**The methods for measuring Lp(a) is not specified, nor is the rationale for the 30 g/dL cut-off.**

**The results are poorly presented and the Discussion is simplistic and inadequate.**

**Response:** The “Introduction” has been pruned considerably.

The Lp(a) analysis has been mentioned as immuno-turbidometric method. The normal range for Lp(a) has been mentioned as 5-30 mg/dl.

The results and discussion has been re-written.

Sincerely

Jaideep

Dr Jaideep C Menon

Amrita Institute of Medical Sciences

Kochi, Kerala, INDIA
