# Supplementary material for: Association of lipoprotein (a) with coronary artery disease in a South Asian population: A case-control study
Source: PLoS One. 2022 May 3;17(5):e0267807. doi: 10.1371/journal.pone.0267807 (PMC9064091; doi:10.1371/journal.pone.0267807)
Supplement: Attachment — Submitted filename: Comment_HYH.docx [file pone.0267807.s003.docx]

***Manuscript Number:*** *PONE-D-21-20756*

***Lipoprotein(a) as a risk factor for cardiovascular disease in a South Indian population: A case control study***

PLOS ONE

Thank you for the opportunity to review this manuscript. The manuscript identified lipoprotein(a) as a risk factor (predictor?) for CVD, particularly those aged below 50 years. Overall, the manuscript is good in its concept and will be of interest to those involved in CVD prevention and management. However, I have major issues in the novelty of the study, methodological issues and results that need to be addressed critically. Specific comments and questions are provided below.

**General**

1. The present manuscript reported on a topic that has been studied previously in India as well as other parts of the world. Many studies, both predictive and causal, have already been published on this topic and found somewhat similar results, indicating LP (a) is an independent risk factor of CAD. On paragraph 6 of the background, several studies have been mentioned from India, which showed more or less consistent results. However, with the abundance of such studies, the motivation to conduct this study is not well justified. What makes this study different from the others in terms of study population, methodology, approach, or context other than a bit larger sample? The authors need to explain why their study was necessary, and what it adds to common knowledge.
2. It is essential to clearly state whether this is a ‘prediction’ study that identifies predictors of CAD or ‘causal’ which needs controlling for confounding and adjustment for selection biases. Based on the description from the introduction and method section, the aim of this study looks more of causal, i.e., means to identify the role of LP (a) as a risk for CAD. However, in the analysis and result section, the authors described the ROC curve, which is completely prediction (discriminatory potential) of LP(a) for CAD, means…confounding is not an issue (Grobbee and Hoes 2014). Moreover, in paragraph 4 of the discussion, the authors emphasize LP(a) to be considered to reclassify patients, which is completely prediction in nature. Thus, the authors should explicitly describe whether the aim is prediction or causal and descriptions should be consistent throughout the manuscript. In addition, if the aim is causal, there are several biochemical parameters (lipid profile) that need to be controlled to claim that LP(a) is an independent risk factor of CAD.

**Title**

3. The title refers to cardiovascular diseases, which includes several categories, which is not limited to coronary artery diseases (CAD). However, the study is specifically for CAD. There are several risk factors which are limited to specific CVD types. If the outcome is CVD, controls are also CVD patients other than CAD. Thus, I would suggest limiting the title specific to CAD, which is the focus of this study.

**Background**

4. Introduction, paragraph 4: the first two descriptions do not have references.
5. The role of paragraph 7 of the background is not clear or not well placed in the background.

**Methods**

6. How the number of cases and control (the ratio) was determined? It is an unbalanced case to control ratio.
7. Controls were supraventricular tachycardia (SVT), atrial fibrillation, atypical chest pain with a normal coronary angiogram. First, these cardiac conditions might be related to the main exposure of interest, i.e. LP(a). This may underestimate the association between LP(a) and CAD. The study would have benefited from multiple control groups. Would it have been better to compare the cases with healthy counterparts in addition to these cases?
8. Ethics statement: It is not clear how this study is exempted from ethical review. It is acceptable that consent might not be required as it is a hospital record. However, it doesn’t necessarily mean ethical review is not required.

**Analysis**

9. In the analysis section, the authors described that Lp(a) is not normally distributed. However, in the result section of the abstract and the main document, LP(a) is described using mean (SD). This is evidenced by table 4 of the result section, which indicates huge variation in mean and median. The authors described that they used log-transformation. Nevertheless, throughout the manuscript, raw values of LP(a) are reported. Thus, the authors should revise the analysis and results accordingly.
10. Receiver operating characteristic curve (ROC) analysis was used to obtain the predictive accuracy of Lp(a) by disease status. What is the importance of ROC…if the study aim is causal.
11. LP(a) is continuous variable and treated as continuous in one occasion and as categorical in another place. It is better to make it consistent based on the prior hypothesis.
12. Table 1 and 2: ‘lipoprotein’, which specific type of lipoprotein?
13. It is puzzling that the main exposure of interest (Lipoprotein) is not included in the multivariable analysis for ‘all ages’.

**Results**

14. The authors described that “*We found that Lp(a) was able to prediction accuracy of Lp(a) was 57% as compared to 62% for diabetes and 70% for tobacco use (Table 5). The ROC curve drawn with these variables in the model gives a predictive probability of about 80%*”. This indicates that the predictive capacity of LP(a) is very low…only 7% over just tossing a coin. It would be better to see the added value of LP(a) apart from other predictors including, tobacco, diabetes, BP, BMI, etc.…

**Discussion**

15. Last paragraph of the discussion section: “*Given that Lp(a) values are stable from the first decade of life and considering that Lp(a) values are genetically determined, Lp(a) should be considered for risk stratification in families with premature CAD and or with high Lp(a) levels in the family*”. Based on this description, the aim of this study is prognostication in its nature, i.e. to evaluate the prognostic performance of LP(a) in predicting CAD. If so, the AUC is only 57%, which is very low, a bit higher than chance. If the aim is not to see the causal role of LP(a) so as to guide preventive interventions, I would suggest restructuring the whole manuscript as a prognostic research. The theoretical design and approach to statistical analysis is different for causal and prediction research.

**Minor issues**

- In general, the manuscript doesn’t have page and line numbers, which make the review process difficult to refer to specific descriptions.
- Be consistent in the use of decimal points

**References**

Grobbee, D. E. and A. W. Hoes (2014). Clinical epidemiology: principles, methods, and applications for clinical research, Jones & Bartlett Publishers.
