# Supplementary material for: Association of lipoprotein (a) with coronary artery disease in a South Asian population: A case-control study
Source: PLoS One. 2022 May 3;17(5):e0267807. doi: 10.1371/journal.pone.0267807 (PMC9064091; doi:10.1371/journal.pone.0267807)
Supplement: Attachment — Submitted filename: Table 7.docx [file pone.0267807.s005.docx]

**Table 7: Summary of case-control studies regarding importance of Lp(a) in CAD in India**

| **First Author** | **Year** | **Journal** | **Cases Lp(a) (n)** | **Controls Lp(a) (n)** | **Comment** |
| --- | --- | --- | --- | --- | --- |
| Vashisht S | 1992 | Indian Heart J. 44(4):223-6 | Lp(a)+++ (760)** | Lp(a)+ (167)** | Clinical CAD |
| Gupta R | 1996 | Int J Cardiol. 57(3):265-70 | 26.8±22.1 (77) | 15.1+14.6 (24) | Angiographic study |
| Mohan V | 1998 | Diabetes Care. 21(11):1819-23 | 24.6 (100)* | 15.1 (100), 19.4 (100)* | Diabetic CAD |
| Singh S | 1999 | J Assoc Phys Ind. 47(12):1157-60 | (222) | (67) | Post MI |
| Gupta R | 2000 | Indian Heart J. 52(4):407-10 | 11.9±2.8 (48)^#^ | 6.7±3.4 (23)^#^ | Recent CAD <60y |
| Gambhir JK | 2000 | Indian Heart J. 52(4):511-5 | 35.0±32.4 (50) | 20.3±17.0 (50) | Young CAD <40y |
| Govindraju V | 2003 | J Ind Med Assoc. 101(8):458-60 | 32.2±1.4 (300) | 30.0±2.6 (200) | Stable CAD |
| Rajasekhar D | 2004 | Ind J Clin Biochem. 19(2):53-9 | 24.8±19.0 (151) | 16.0±17.5 (49) | Stable CAD |
| Wadhwa A | 2013 | J Assoc Phys Ind. 61(6):384-6 | 38.7±26.2 (40) | 26.2±20.5 (40) | Young MI ≤45y |
| Yusuf J | 2014 | Indian Heart J. 66(3):272-9 | 30.3 (450)* | 20.0 (150)* |  |
| Bansal SK | 2015 | J Clin Diagn Res. 9(11):BC07-11 | 43.2±10.2 (30) | 17.6±3.2 (30) | Stable CAD <60y |
| Ramesh G | 2018 | Interv Med Appl Sci. 10(2):65-9 | 33.8±23.7 (51) | 19.7±10.4 (51) | Young MI ≤45y |
| Gupta MD | 2018 | Ind Heart J. 70(S3):S146-56 | 37.3±4.1 (125) | 27.3±3.4 (103) | Young MI ≤35y |
| Hanif S | 2019 | Pak J Med Sci. 35(6):1718-23 | 47.0±45.5 (90) | 29.7±23.1 (90) | Young MI ≤45y |
| *median value; ^#^geometric mean; CAD coronary artery disease; MI myocardial infarction; **immunofluorescence technique | | | | | |
