# Supplementary material for: Association of lipoprotein (a) with coronary artery disease in a South Asian population: A case-control study
Source: PLoS One. 2022 May 3;17(5):e0267807. doi: 10.1371/journal.pone.0267807 (PMC9064091; doi:10.1371/journal.pone.0267807)
Supplement: Attachment — Submitted filename: Response to the reviewers comments2.docx [file pone.0267807.s006.docx]

**Authors Response to the reviewers comments**

Reviewer #1: Thanks for submitting a revised version of this article. The manuscript is now much better, but some deficiencies remain.

Response: We thank the reviewer for his encouraging remarks.

Comment 1. The authors have log transformed the Lp(a) values and then performed the quantitative analyses. This is also an appropriate method, although I believe that using non-parametric comparisons such as median test for 2 group and Kruskal-Wallis test for multiple group comparisons are better methods. This should be discussed in the limitation sections of the article.

Response: We have reported the median Lp(a) values and used the median test for comparison in the revised version of the manuscript. (Line 23-26, page 3 and Table 4 page 11)

Comment 2. The interquartile ranges are typically presented as median (25-75 interquartile range, IQR), e.g. 55 (40-70). Nowhere the authors have presented data in this format. Moreover, the authors have reported mean levels of Lp(a) without any measurement of dispersion in the abstract, text and tables. All these should be revised.

Response: We have reported the (25-75 IQR) in table 4 page 11 of the revised manuscript.

Comment 3. The discussion section is still too long and instead of discussing the findings of the article and comparing these with previous Indian and international studies, the authors focus on implications of the findings. Please modify.

Response: The discussion section has been suitably altered comparing between previous studies and the present one. The implications of our study have been removed from the section.

4. Studies on Lp(a) from India are listed below. There are many such studies and should be referenced.

Response: Thank you very much, all the references have been included and cited.

Reviewer #2: Manuscript Number: PONE-D-21-20756R1
Lipoprotein(a) as a risk factor for coronary artery disease in a South Asian population: A case control study
PLOS ONE

Thank you again for the opportunity to review this manuscript. However, I still have major issues that need to be addressed critically. Specific comments and questions are provided below.
Response: We thank the reviewer for his comments. We have responded to each one of his comments in the revised manuscript

Comment 1. The authors claim that it is a prediction study not causal or risk factor analysis. However, the title still reads as “…as a risk factor….”. The theoretical design, the approach to data analysis and interpretation of the study varies significantly for prediction and causal research. The description in the abstract and the whole body of the manuscript still reads as ‘…risk factor.’. In prediction, individual ORs do not have interpretive value. Prediction is not necessarily risk factor identification.

Response: Thanks for the detailed observation. We have studied the association of Lp(a) with CAD in the presence of other known factors. We have changed the title of the manuscript now.

Comment 2. I still suggest authors consider evaluating the added value of LP(a) in predicting CAD along with other predictors such as tobacco, DM, etc.…. Whether statistically significant or not, AUC of 60% has minimal clinical importance. It is better to develop a multivariable prediction model with and without LP(a) and assess the added value.

Response: We thank the reviewer for his valuable suggestions. We have now added another table for logistic regression for individuals below 50 years(Table 3) in the revised manuscript in page 10. Two models Model 1 and Model 2 have been developed. Model 1 has CAD has the dependent variable and gender(female/male), diabetes(yes/no), hypertension(yes/no), tobacco use (yes/no), alcohol use (yes/no) as predictor variables and Model 2 has CAD has the dependent variable and gender(female/male), diabetes(yes/no), hypertension(yes/no), tobacco use (yes/no), alcohol use (yes/no) and Lp(a)(<50 and >=50) as predictors. The deviance between Model 1 and Model 2 was 9.82 which is statistically significant at p<0.05 when compared with χ2 with 1 d.f. Thus, Model 2 with Lp(a) is a better predictor model for the disease as compared to Model 1 without it, indicating that Lp(a) is an independent predictor for the disease in addition to the established risk factors viz. presence of diabetes and tobacco use

Comment 3. Although LP(a) is not normally distributed, using mean with SD is not justified enough.

Response: We have not reported mean Lp(a) anywhere in the revised manuscript now.

Comment 4. Whether significant or not on the univariable analysis, the main predictor of interest (LP (a)) should be included in the multivariable analysis.

Response: We have now revised Table 1 for all individuals after including Lp(a) as a predictor in the multivariable analysis.

Comment 5. I commend the authors for uploading the data used to produce this manuscript. It helps for open science. Looking in to the supporting information (Data), there are lots of missing values. However, there is no description on the missing data. Did the authors check for the missingness pattern? How did they manage it?

Response: We had uploaded a revised data sheet with the variables used for the analysis.

Comment6. In addition, the outcome variable and predictors are not clearly labeled in the data. I suggest authors make it more clear and transparent.

Response: A revised data sheet is being uploaded with clear labels

Comment 7. In spite of the comment in round 1 to add for line numbers, the manuscript still doesn’t have page and line numbers, which make the review process difficult to refer to specific descriptions. As far as I understand, the author guideline requests to put line numbers to ease the review process.

Response: Thanks for the suggestions. We have now inserted the page numbers and the line numbers now in the revised manuscript.
