# Supplementary material for: Association of lipoprotein (a) with coronary artery disease in a South Asian population: A case-control study
Source: PLoS One. 2022 May 3;17(5):e0267807. doi: 10.1371/journal.pone.0267807 (PMC9064091; doi:10.1371/journal.pone.0267807)
Supplement: Attachment — Submitted filename: Authors Response to the reviewers comments_R3.docx [file pone.0267807.s007.docx]

**Authors Response to the reviewers comments**

**Reviewer #1:**

1**.** The Tables are very lengthy and provide unnecessary information.

Response: We appreciate the reviewer’s suggestion. The tables have been reduced after removing unnecessary information

2. I would suggest that in each row, in Tables 1 and 2, the authors only retain the Yes (risk factor, positive findings) row and delete the No row. This would make the Tables less cluttered and easier to comprehend.

Response:. We have reduced the ‘No’ categories for each variable. The information in tables 2 and 3 has now been combined into one table.

Reviewer #2: Manuscript Number: PONE-D-21-20756R2 Association of lipoprotein (a) with coronary artery disease in a South Asian population: A case control study
PLOS ONE
Thank you once more. The authors addressed most of my concerns. However, I still have issues that need to be addressed. Specific comments and questions are provided below.

Response: We are glad that we have been able to address many of the reviewers concern.

1. The authors’ focus shifted from a kind of prediction research to “Association of lipoprotein (a) with coronary artery disease”. Thus, the study approach, statistical analysis and interpretation of results would also change. There are descriptions in the manuscript that seem for prediction type of research. Here are some of the descriptions in the manuscript that confused risk factor identification research to prediction…

•First sentence of the discussion section still reads as “The study results suggest that Lp(a) has a modest predictive accuracy for CAD especially in subjects ≤50 years of age.”

Response: We have now rewritten the first paragraph of the discussion section

•The first sentence of summary (page 13, line 13-14) also phrased as “In summary our study suggests that Lp(a) is an important predictor for CAD especially in the age group < 50 years.”

Response: We have rewritten the first sentence of the summary section

•The conclusion section of the abstract.

Response: Response: We have rewritten the conclusion section of the abstract.

•Abstract line 13

Response: We have rewritten the abstract line 13

•Analysis section, page 7 line 3 to 8. This description of analysis is still on prediction.

Response: The analysis section has been rewritten as an association study

•Result page 10, lines 5 to 9: these descriptions are for prediction type of research.

Response: The results section has been rewritten as an association study
